# A comparison of risk factors for breech presentation in preterm and term labor: a nationwide, population-based case–control study

**DOI:** 10.1007/s00404-019-05385-5

**Published:** 2019-11-18

**Authors:** Anna E. Toijonen, Seppo T. Heinonen, Mika V. M. Gissler, Georg Macharey

**Affiliations:** 1 Department of Obstetrics and Gynecology, University Hospital (HUS), University of Helsinki, Haartmaninkatu 2, 00290 Helsinki, Finland; 2 National Institute for Health and Welfare (THL), Helsinki, Finland; 3 School of Medicine, University of Helsinki, Helsinki, Finland

**Keywords:** Breech presentation, Risk factor, Preterm delivery, Preterm labor

## Abstract

**Purpose:**

To determine if the common risks for breech presentation at term labor are also eligible in preterm labor.

**Methods:**

A Finnish cross-sectional study included 737,788 singleton births (24–42 gestational weeks) during 2004–2014. A multivariable logistic regression analysis was used to calculate the risks of breech presentation.

**Results:**

The incidence of breech presentation at delivery decreased from 23.5% in pregnancy weeks 24–27 to 2.5% in term pregnancies. In gestational weeks 24–27, preterm premature rupture of membranes was associated with breech presentation. In 28–31 gestational weeks, breech presentation was associated with maternal pre-eclampsia/hypertension, preterm premature rupture of membranes, and fetal birth weight below the tenth percentile. In gestational weeks 32–36, the risks were advanced maternal age, nulliparity, previous cesarean section, preterm premature rupture of membranes, oligohydramnios, birth weight below the tenth percentile, female sex, and congenital anomaly. In term pregnancies, breech presentation was associated with advanced maternal age, nulliparity, maternal hypothyroidism, pre-gestational diabetes, placenta praevia, premature rupture of membranes, oligohydramnios, congenital anomaly, female sex, and birth weight below the tenth percentile.

**Conclusion:**

Breech presentation in preterm labor is associated with obstetric risk factors compared to cephalic presentation. These risks decrease linearly with the gestational age. In moderate to late preterm delivery, breech presentation is a high-risk state and some obstetric risk factors are yet visible in early preterm delivery. Breech presentation in extremely preterm deliveries has, with the exception of preterm premature rupture of membranes, similar clinical risk profiles as in cephalic presentation.

## Introduction

The prevalence of breech presentation at delivery decreases with increasing gestational age. At 28 pregnancy weeks, every fifth fetus lies in the breech presentation and in term pregnancies, less than 4% of all singleton fetuses are in breech presentation at delivery [1, 2]. Most likely this is due to a lack of fetal movements [3] or an incomplete fetal rotation, since the possibility of a spontaneous rotation declines with increasing gestational age. Consequently, preterm labor itself is often associated with breech presentation at delivery, since the fetus was not yet able to rotate [4–9]. This fact makes preterm labor as one of the strongest risk factors for breech presentation.

Vaginal breech delivery in term pregnancies is not only associated with poorer perinatal outcomes compared to vaginal delivery with a fetus in cephalic presentation [6, 10, 11], but also it is debated whether the cause of breech presentation itself is a risk for adverse peri- and neonatal outcomes [3, 12, 13]. Several fetal and maternal features, such as fetal growth restriction, congenital anomaly, oligohydramnios, gestational diabetes, and previous cesarean section, are linked to a higher risk of breech presentation at term, and, furthermore, are associated with an increased risk for adverse perinatal outcomes [3–5, 8, 9, 14–17].

The literature lacks studies on the risk factors of breech presentation in preterm pregnancies. It remains unclear whether breech presentation at preterm labor is only caused by the incomplete fetal rotation, or whether breech presentation in preterm labor is also associated with other obstetric risk factors. Most of the studies reviewing risk factors for breech presentation focus on term pregnancies. Our hypothesis is that breech presentation in preterm deliveries is, besides preterm pregnancy itself, associated with other risk factors similar to breech presentation at term. We aim to compare the risks of preterm breech presentation to those in cephalic presentation by gestational age. Such information would be valuable in the risk stratification of breech deliveries by gestational age.

## Materials and methods

We conducted a retrospective population-based cross-sectional study. The population included all the singleton preterm and term births, from January 2004 to December 2014 in Finland. The data were collected from the national medical birth register and the hospital discharge register, maintained by the National Institute for Health and Welfare. All Finnish maternity hospitals are obligated to contribute clinical data on births from 22 weeks or birth weight of 500 g to the register. All newborn infants are examined by a pediatrician and given a personal identification number that can be traced in the case of perinatal mortality or morbidity. The hospital discharge register contains information on all surgical procedures and diagnoses (International Statistical Classification of Diseases and Related Health Problems 10th Revision, ICD-10) in all inpatient care and outpatient care in public hospitals.

Authorization to use the data was obtained from the National Institute for Health and Welfare as required by the national data protection law in Finland (reference number THL/652/5.05.00/2017).

**Fig. 1:**
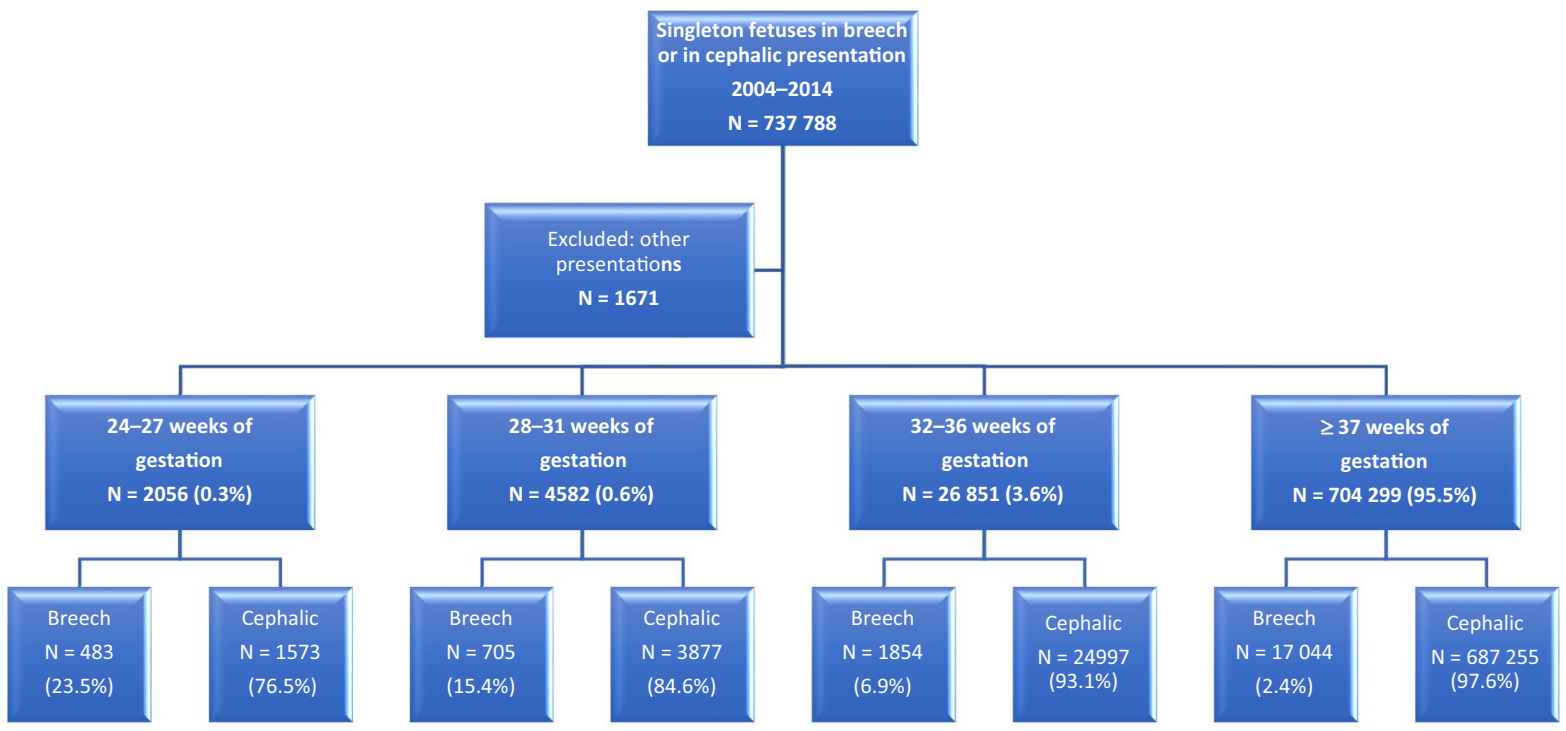
Breech presentation for singleton pregnancies during the period of 2004–2014 in Finland

The study population included all the women with a singleton fetus in breech presentation at the time of delivery. The control group included all the women with a singleton fetus in cephalic presentation at delivery. Other presentations were excluded from the study (*N* = 1671) (Fig. 1). Gestational age was determined according to early ultrasonographic measurement which is routinely performed in Finland and it encompasses over 95% of the mothers, or if not available, to the last menstrual period. We excluded neonates delivered before 24 weeks of gestation and birth weight of less than 500 g, because the lower viability may have influenced the mode of the delivery or the outcome. The study population was divided into four categories according to the World Health Organization (WHO) definitions of preterm and term deliveries. WHO defines preterm birth as a fetus born alive before 37 completed weeks of pregnancy. WHO recommends sub-categories of preterm birth, based on gestational age, as extremely preterm (less than 28 pregnancy weeks), very preterm (28–32 pregnancy weeks), and moderate to late preterm (32–37 pregnancy weeks).

In our study, we assessed four factors that may be associated with breech presentation based on prior reports [3–5, 14, 17–20]. These factors were: maternal age below 25 and 35 years or more, smoking, pre-pregnancy body mass index (BMI) over 30, and in vitro fertilization. The following factors were also analyzed: nulliparity, more than three previous deliveries, and history of cesarean section. The obstetric risk factors including maternal hypo- or hyperthyroidism (ICD-10 E03, E05), gestational diabetes (ICD-10 O24.4) and other diabetes treated with insulin (ICD-10 O24.0), arterial hypertension or pre-eclampsia (ICD-10 O13, O14), and maternal care for (suspected) damage to fetus by alcohol or drugs (ICD-10 O35.4, O35.5) were assessed in the analysis. The variables that were also included in the analysis were: oligohydramnios (ICD-10 O41.0), placenta praevia (ICD-10 O44), placental abruption (ICD-10 O45), preterm premature rupture of membranes (PPROM) (ICD-10 O42), infant sex, fetal birth weight below the tenth percentile, fetuses with birth weight above the 97th percentile, and fetal congenital anomalies as defined in the register of congenital malformations.

The babies born in breech presentation from the four study groups were compared with the babies born in cephalic presentation with the equal gestational age, according to WHO classification. The calculations were performed using SPSS 19. Statistical differences in categorical variables were evaluated with the Chi-squared test or Fisher’s exact test when appropriate. We calculated odds ratios (ORs) with corresponding 95% confidence intervals (CIs) using binary logistic regression. Each study group was separately adjusted, according to gestational age at delivery, defined by WHO. The adjustment for the risk factors was done by multivariable logistic regression model for all variables. Differences were deemed to be statistically significant with *P* value < 0.05.

## Results

This analysis includes 737,788 singleton births, from these 20,086 were in breech presentation at the time of delivery. Out of all deliveries, 33,489 infants were born preterm. The prevalence of breech presentation at delivery decreased with the increase of the gestational age: 23.5% in extremely preterm delivery, 15.4% very preterm deliveries, and 6.7% in moderate to late preterm deliveries. At term, the prevalence of breech presentation at delivery was 2.5% (Fig. 1).

**Fig. 2:**
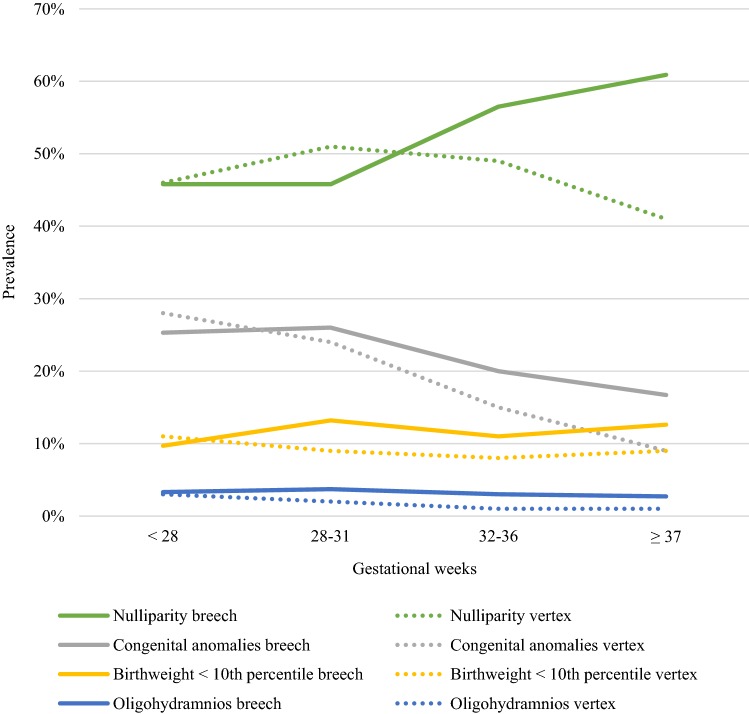
Prevalence of obstetric risk factors for breech presentation compared to cephalic by gestational age. *PPROM* preterm premature rupture of membranes, *PROM* premature rupture of membranes

**Fig. 3:**
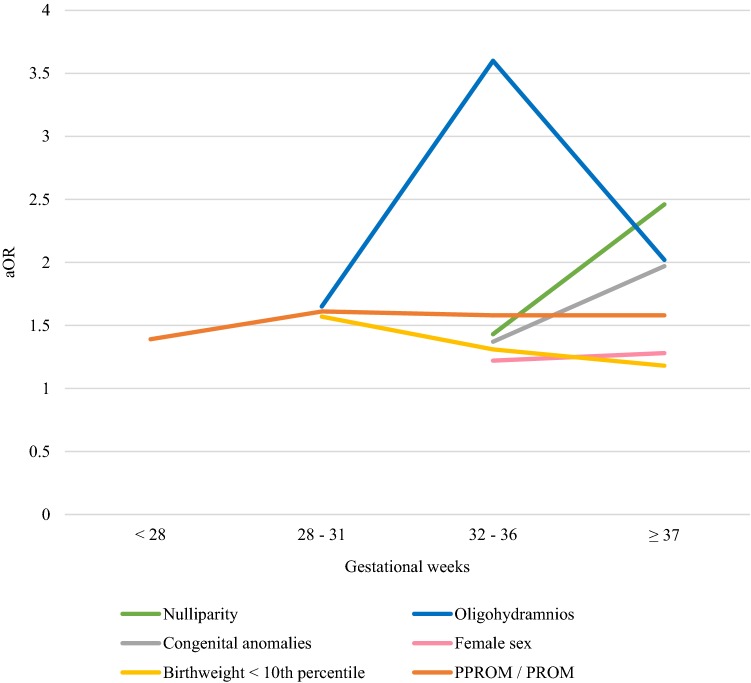
Obstetric risk factors for breech presentation with adjusted odds ratios by gestational age. *PPROM* preterm premature rupture of membranes, *PROM* premature rupture of membranes, *aOR* adjusted odds ratio

**Fig. 4:**
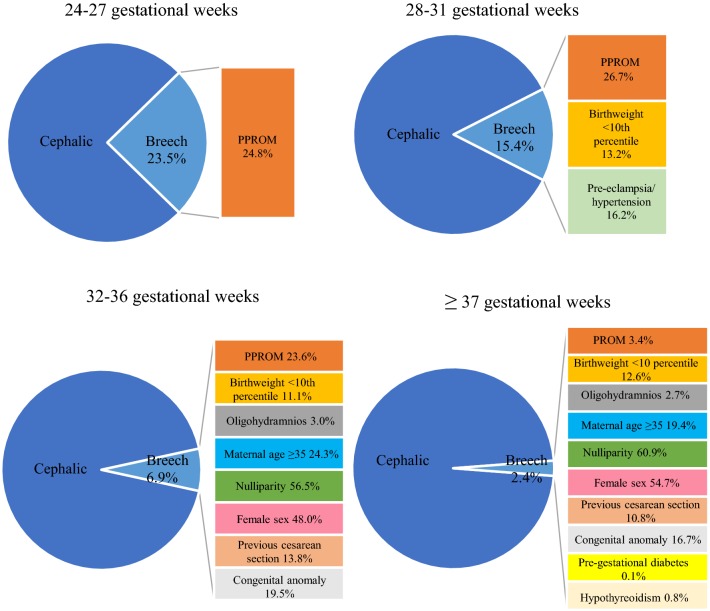
The determinants of breech presentation by gestational age. *PPROM* preterm premature rupture of membranes, *PROM* premature rupture of membranes

From all deliveries, 2056 fetuses were born extremely preterm (24 + 0 to 27 + 6 gestational weeks). The differences in the possible risk factors for breech presentation at delivery were higher odds of PPROM (aOR 1.39, 95% CI 1.08–1.79, *P* = 0.010) and a lower risk of placental abruption (aOR 0.59, 95% CI 0.36–0.98, *P* = 0.040). No statistically significant differences were observed for the other factors (Table 1, Figs. 1, 2, 3, 4).

**Table 1:** Unadjusted and adjusted odds ratios for risk factors in singleton extremely preterm 24 + 0 to 27 + 6 weeks of gestational age fetuses in breech and in cephalic presentations during 2004–2014 in Finland

24–27 Weeks of gestation	Breech (*N* = 483)	Cephalic (*N* = 1573)	*P* value	Odds ratio (95% Cl)	Adjusted odds ratio (95% Cl)
Maternal age < 25	17 (3.5%)	37 (2.4%)	0.153	1.51 (0.84–2.71)	1.56 (0.85–2.84)
Maternal age ≥ 35	129 (26.7%)	438 (27.8%)	0.606	0.94 (0.75–1.19)	0.94 (0.73–1.20)
Smoking	77 (15.9%)	251 (16.0%)	0.934	1 (0.76–1.32)	0.98 (0.74–1.30)
Maternal BMI ≥ 25	78 (16.10%)	262 (16.7%)	0.499	0.96 (0.76–1.32)	0.89 (0.62–1.27)
Maternal BMI ≥ 30	33 (6.8%)	104 (6.6%)	0.898	1.04 (0.69–1.55)	1.03 (0.61–1.75)
Nulliparity	221 (45.8%)	727 (46.2%)	0.409	0.98 (0.80–1.20)	0.91 (0.71–1.16)
Parity ≥ 3	66 (13.7%)	220 (14.0%)	0.983	0.97 (0.72–1.31)	1.01 (0.73–1.40)
Maternal hypothyroidism	6 (1.2%)	9 (0.6%)	0.159	2.19 (0.77–6.17)	2.15 (0.74–6.22)
Maternal hyperthyroidism	1 (0.2%)	3 (0.2%)	0.783	1.09 (0.11–10.46)	1.38 (0.14–13.62)
Pre-gestational diabetes treated with insulin	2 (0.4%)	6 (0.4%)	0.577	1.09 (0.22–5.40)	1.27 (0.55–2.96)
Gestational diabetes	20 (4.1%)	48 (3.1%)	0.222	1.37 (0.81–2.34)	1.42 (0.81–2.49)
Pre-eclampsia/hypertension	34 (7.0%)	84 (5.3%)	0.083	1.34 (0.89–2.03)	1.46 (0.95–2.24)
Previous cesarean section	64 (13.3%)	232 (14.7%)	0.294	0.88 (0.66–1.19)	0.85 (0.61–1.17)
IVF	17 (3.5%)	64 (4.1%)	0.828	0.86 (0.50–1.48)	0.94 (0.53–1.65)
Maternal care for (suspected) damage to fetus by alcohol/drugs	0 (0.0%)	3 (0.2%)	0.971		
Placenta praevia	9 (1.9%)	29 (1.8%)	0.981	1.01 (0.48–2.15)	1.01 (0.47–2.18)
Placental abruption	20 (4.1%)	101 (6.4%)	0.040	0.63 (0.39–1.03)	0.59 (0.36–0.98)
PPROM	120 (24.8%)	308 (19.6%)	0.010	1.36 (1.07–1.73)	1. 39 (1.08–1.79)
Oligohydramnios	16 (3.3%)	45 (2.9%)	0.625	1.16 (0.65–2.08)	1.16 (0.64–2.11)
Congenital anomaly	122 (25.3%)	435 (27.7%)	0.242	0.88 (0.70–1.12)	0.87 (0.68–1.10)
Female sex	234 (48.4%)	734 (46.7%)	0.584	1.07 (0.88–1.32)	1.06 (0.86–1.30)
Birthweight < 10th percentile	47 (9.7%)	174 (11.1%)	0.486	0.87 (0.62–1.22)	1.16 (0.76–1.78)
Birthweight > 97th percentile	4 (0.8%)	15 (1.0%)	0.905	0.87 (0.29–2.63)	0.94 (0.30–2.89)

*BMI* body mass index, *IVF* in vitro fertilization, maternal intoxication, *PPROM* preterm premature rupture of membranes

The group of very preterm deliveries (28 + 0 to 31 + 6 gestational weeks) included 4582 singleton newborns. Breech presentation at delivery was associated with PPROM (aOR 1.61, 95% CI 1.32–1.96, *P* < 0.001), oligohydramnios (aOR 1.65, 95% CI 1.03–2.64, *P* = 0.038), fetal birth weight below the tenth percentile (aOR 1.57, 95% CI 1.19–2.08, *P* = 0.002), and maternal pre-eclampsia and arterial hypertension (aOR 1.31, 95% CI 1.04–1.66, *P* = 0.023). Details of risk factors in very preterm breech deliveries are described in Table 2. The risk of placenta praevia as well as having a birth weight above the 97th percentile was lower in pregnancies with fetuses in breech rather than in cephalic presentation (Table 2, Figs. 2, 3, 4).

**Table 2:** Unadjusted and adjusted odds ratios for risk factors in singleton very preterm 28 + 0 to 31 + 6 weeks of gestational age fetuses in breech and in cephalic presentations during 2004–2014 in Finland

28–31 Weeks of gestation	Breech (*N* = 705)	Cephalic (*N* = 3877)	*P* value	Odds ratio (95% Cl)	Adjusted odds ratio (95% CI)
Maternal age < 25	10 (1.4%)	108 (2.8%)	< 0.001	0.50 (0.26–0.96)	0.57 (0.29–1.10)
Maternal age ≥ 35	182 (25.8%)	954 (24.6%)	0.095	1.07 (0.89–1.28)	0.97 (0.80–1.18)
Smoking	105 (14.9%)	700 (18.1%)	0.064	0.79 (0.64–0.99)	0.81 (0.64–1.01)
Maternal BMI ≥ 25	109 (15.5%)	532 (13.7%)	0.124	1.15 (0.92–1.44)	1.24 (0.94–1.63)
Maternal BMI ≥ 30	33 (4.7%)	207 (5.3%)	0.053	0.87 (0.60–1.27)	0.64 (0.41–1.01)
Nulliparity	323 (45.8%)	1972 (50.9%)	0.121	0.82 (0.70–0.96)	0.86 (0.71–1.04)
Parity ≥ 3	96 (13.6%)	412 (10.6%)	0.202	1.33 (1.04–1.68)	1.19 (0.91–1.54)
Maternal hypothyroidism	8 (1.1%)	35 (0.9%)	0.888	1.26 (0.58–2.73)	1.06 (0.48–2.34)
Maternal hyperthyroidism	3 (0.4%)	6 (0.2%)	0.227	2.76 (0.69–11.05)	2.38 (0.58–9.72)
Pre-gestational diabetes treated with insulin	5 (0.7%)	16 (0.4%)	0.155	1.72 (0.63–4.72)	1.39 (0.88–2.18)
Gestational diabetes	59 (8.4%)	248 (6.4%)	0.086	1.34 (0.99–1.80)	1.31 (0.96–1.79)
Pre-eclampsia/hypertension	114 (16.2%)	514 (13.3%)	0.023	1.26 (1.01–1.57)	1.31 (1.04–1.66)
Previous cesarean section	128 (18.2%)	519 (15.2%)	0.443	1.23 (1.00–1.52)	1.10 (0.86–1.40)
IVF	22 (3.1%)	169 (4.4%)	0.122	0.71 (0.45–1.11)	0.68 (0.41–1.11)
Maternal care for (suspected) damage to fetus by alcohol/drugs	0 (0.0%)	9 (0.2%)	0.973		
Placenta praevia	9 (1.3%)	133 (3.4%)	0.004	0.36 (0.18–0.72)	0.36 (0.18–0.72)
Placental abruption	32 (4.5%)	232 (6.0%)	0.225	0.75 (0.51–1.09)	0.79 (0.54–1.16)
PPROM	188 (26.7%)	764 (19.7%)	< 0.001	1.48 (1.23–1.78)	1.61 (1.32–1.96)
Oligohydramnios	26 (3.7%)	73 (1.9%)	0.038	2.00 (1.27–3.15)	1.65 (1.03–2.64)
Congenital anomaly	183 (26.0%)	946 (24.4%)	0.453	1.09 (0.90–1.31)	1.08 (0.89–1.30)
Female sex	315 (44.7%)	1739 (44.9%)	0.924	0.99 (0.84–1.17)	0.99 (0.84–1.17)
Birthweight < 10th percentile	93 (13.2%)	348 (9.0%)	0.002	1.54 (1.21–1.97)	1.57 (1.19–2.08)
Birthweight > 97th percentile	8 (1.1%)	97 (2.5%)	0.022	0.45 (0.22–0.92)	0.42 (0.20–0.89)

*BMI* body mass index, *IVF* in vitro fertilization, *PPROM* preterm premature rupture of membranes

The moderate to late preterm delivery group (32 + 0 to 36 + 6 gestational weeks) included 26,851 deliveries. Breech presentation in moderate to late preterm deliveries was associated with older maternal age (maternal age 35 years or more aOR 1.24, 95% CI 1.10–1.39, *P* < 0.001), nullipara (aOR 1.43, 95% CI 1.27–1.60, *P* < 0.001), maternal BMI less than 25 (maternal BMI ≥ 25 aOR 0.75, 95% CI 0.62–0.91, *P* = 0.004), previous cesarean section (aOR 1.31, 95% CI 1.12–1.53, *P* < 0.001), female sex (aOR 1.22, 95% CI 1.11–1.34, *P* < 0.001), congenital anomaly (aOR 1.37, 95% CI 1.22–1.55, *P* < 0.001), fetal birth weight below the tenth percentile (aOR 1.31, 95% CI 1.10–1.56, *P* = 0.003), oligohydramnios (aOR 3.60, 95% CI 2.63–4.92, *P* < 0.001), and PPROM (aOR 1.58, 95% CI 1.41–1.78, *P* < 0.001). Breech presentation decreased the odds of having a fetus with birth weight above the 97th percentile (aOR 0.60, 95% CI 0.42–0.85, *P* = 0.004) (Table 3, Figs. 2, 3, 4).

**Table 3:** Unadjusted and adjusted odds ratios for risk factors in singleton moderate to late preterm 32 + 0 to 36 + 6 weeks of gestational age fetuses in breech and in cephalic presentations during 2004–2014 in Finland

32–36 Weeks of gestation	Breech (*N* = 1854)	Cephalic (*N* = 24 997)	*P* value	Odds ratio (95% Cl)	Adjusted odds ratio (95% CI)
Maternal age < 25	39 (2.1%)	741 (3.0%)	0.020	0.70 (0.51–0.97)	0.68 (0.48–0.94)
Maternal age ≥ 35	451 (24.3%)	5409 (21.6%)	< 0.001	1.16 (1.04–1.30)	1.24 (1.10–1.39)
Smoking	293 (15.8%)	4426 (17.7%)	0.139	0.87 (0.77–0.99)	0.91 (0.79–1.03)
Maternal BMI ≥ 25	202 (10.9%)	3359 (13.4%)	0.004	0.79 (0.68–0.92)	0.75 (0.62–0.91)
Maternal BMI ≥ 30	80 (4.3%)	1175 (4.7%)	0.120	0.91 (0.73–1.15)	1.26 (0.94–1.69)
Nulliparity	1048 (56.5%)	12,235 (48.9%)	< 0.001	1.36 (1.23–1.49)	1.43 (1.27–1.60)
Parity ≥ 3	158 (8.5%)	2665 (10.7%)	0.134	0.78 (0.66–0.92)	0.87 (0.73–1.04)
Maternal hypothyroidism	21 (1.1%)	259 (1.0%)	0.360	1.09 (0.70–1.71)	1.24 (0.78–1.96)
Maternal hyperthyroidism	6 (0.3%)	48 (0.2%)	0.100	1.69 (0.72–3.95)	2.06 (0.87–4.87)
Pre-gestational diabetes treated with insulin	5 (0.3%)	118 (0.5%)	0.066	0.57 (0.23–1.40)	0.76 (0.57–1.02)
Gestational diabetes	159 (8.6%)	2481 (9.9%)	0.099	0.85 (0.72–1.01)	0.86 (0.72–1.03)
Pre-eclampsia/hypertension	161 (8.7%)	2232 (8.9%)	0.394	0.97 (0.82–1.15)	0.93 (0.78–1.10)
Previous cesarean section	255 (13.8%)	3423 (13.7%)	< 0.001	1.01 (0.88–1.15)	1.31 (1.12–1.53)
IVF	75 (4.0%)	900 (3.6%)	0.854	1.13 (0.89–1.44)	0.98 (0.76–1.25)
Maternal care for (suspected) damage to fetus by alcohol/drugs	3 (0.2%)	39 (0.2%)	0.760	1.04 (0.32–3.36)	0.83 (0.25–2.76)
Placenta praevia	36 (1.9%)	624 (2.5%)	0.240	0.77 (0.55–1.09)	0.81 (0.58–1.15)
Placental abruption	27 (1.5%)	414 (1.7%)	0.763	0.88 (0.59–1.30)	0.94 (0.63–1.40)
PPROM	437 (23.6%)	3968 (15.9%)	< 0.001	1.63 (1.46–1.83)	1.58 (1.41–1.78)
Oligohydramnios	55 (3.0%)	191 (0.8%)	< 0.001	3.97 (2.93–5.38)	3.60 (2.63–4.92)
Congenital anomaly	362 (19.5%)	3690 (14.8%)	< 0.001	1.40 (1.24–1.58)	1.37 (1.22–1.55)
Female sex	890 (48.0%)	10,817 (43.3%)	< 0.001	1.21 (1.10–1.33)	1.22 (1.11–1.34)
Birthweight < 10th percentile	205 (11.1%)	2012 (8.0%)	0.003	1.42 (1.22–1.65)	1.31 (1.10–1.56)
Birthweight > 97th percentile	41 (2.2%)	1162 (4.6%)	0.004	0.46 (0.34–0.64)	0.60 (0.42–0.85)

*BMI* body mass index, *IVF* in vitro fertilization, *PPROM* preterm premature rupture of membranes

The term and post-term group included 704,299 deliveries, among them 17,044 fetuses in breech presentation. The factors associated with breech presentation amongst these were: maternal age of 35 years or more (aOR 1.24, 95% CI 1.19–1.29, *P* < 0.001), nullipara (aOR 2.46, 95% CI 2.37–2.55, *P* < 0.001), maternal BMI less than 25 (BMI ≥ 25 aOR 0.90, 95% CI 0.85–0.96, *P* < 0.001), maternal hypothyroidism (aOR 1.53, 95% CI 1.28–1.82, *P* < 0.001), pre-gestational diabetes treated with insulin (aOR 1.24, 95% CI 1.00–1.53, *P* = 0.049), placenta praevia (aOR 1.45, 95% CI 1.11–1.91, *P* = 0.007), premature rupture of membranes (PROM) (aOR 1.58, 95% CI 1.45–1.72, *P* < 0.001), oligohydramnios (aOR 2.02, 95% CI 1.83–2.22, *P* < 0.001), congenital anomaly (aOR 1.97, 95% CI 1.89–2.06, *P* < 0.001), female sex (aOR 1.28, 95% CI 1.24–1.32, *P* < 0.001), and birth weight below the tenth percentile (aOR 1.18, 95% CI 1.12–1.24, *P* < 0.001) Table 4 includes details for risk factors of term and post-term group (Figs. 2, 3, 4).

**Table 4:** Unadjusted and adjusted odds ratios for risk factors in singleton term pregnancies in breech and in cephalic presentations during 2004–2014 in Finland

≥ 37 Weeks of gestation	Breech (*N* = 17 044)	Cephalic (*N* = 687 255)	*P* value	Odds ratio (95% Cl)	Adjusted odds ratio (95% CI)
Maternal age < 25	304 (1.8%)	15,496 (2.3%)	< 0.001	0.79 (0.70–0.88)	0.57 (0.51–0.64)
Maternal age ≥ 35	3313 (19.4%)	130,687 (19.0%)	< 0.001	1.03 (0.99–1.07)	1.24 (1.19–1.29)
Smoking	2593 (15.2%)	102,333 (14.9%)	0.845	1.03 (0.98–1.07)	1.00 (0.95–1.04)
Maternal BMI ≥ 25	1753 (10.3%)	79,114 (11.5%)	< 0.001	0.88 (0.84–0.93)	0.90 (0.85–0.96)
Maternal BMI ≥ 30	588 (3.4%)	25,854 (3.8%)	0.56	0.91 (0.84–0.99)	1.03 (0.93–1.14)
Nulliparity	10,387 (60.9%)	281,094 (40.9%)	< 0.001	2.25 (2.19–2.33)	2.46 (2.37–2.55)
Parity ≥ 3	910 (5.3%)	68,532 (10.0%)	< 0.001	0.51 (0.48–0.54)	0.75 (0.70–0.81)
Maternal hypothyroidism	131 (0.8%)	3146 (0.5%)	< 0.001	1.68 (1.41–2.01)	1.53 (1.28–1.82)
Maternal hyperthyroidism	22 (0.1%)	634 (0.1%)	0.082	1.40 (0.91–2.14)	1.46 (0.95–2.24)
Pre-gestational diabetes treated with insulin	24 (0.1%)	789 (0.1%)	0.049	1.23 (0.82–1.84)	1.24 (1.00–1.53)
Gestational diabetes	1447 (8.5%)	57,613 (8.4%)	0.418	1.01 (0.96–1.07)	1.02 (0.97–1.08)
Pre-eclampsia/hypertension	600 (3.5%)	21,627 (3.1%)	0.07	1.12 (1.03–1.22)	0.93 (0.85–1.01)
Previous cesarean section	1847 (10.8%)	73,575 (10.7%)	< 0.001	1.01 (0.97–1.06)	1.67 (1.58–1.76)
IVF	483 (2.8%)	14,393 (2.1%)	0.68	1.36 (1.24–1.49)	0.98 (0.89–1.08)
Maternal care for (suspected) damage to fetus by alcohol/drugs	6 (0.0%)	734 (0.1%)	0.001	0.33 (0.15–0.74)	0.27 (0.12–0.60)
Placenta praevia	55 (0.3%)	1418 (0.2%)	0.007	1.57 (1.20–2.05)	1.45 (1.11–1.91)
Placental abruption	23 (0.1%)	995 (0.1%)	0.496	0.93 (0.62–1.41)	0.87 (0.75–1.31)
PROM	582 (3.4%)	12,938 (1.9%)	< 0.001	1.84 (1.69–2.01)	1.58 (1.45–1.72)
Oligohydramnios	453 (2.7%)	7867 (1.1%)	< 0.001	2.36 (2.14–2.60)	2.02 (1.83–2.22)
Congenital anomaly	2846 (16.7%)	62 002 (9.0%)	< 0.001	2.02 (1.94–2.11)	1.97 (1.89–2.06)
Female sex	9321 (54.7%)	336,313 (48.9%)	< 0.001	1.26 (1.22–1.30)	1.28 (1.24–1.32)
Birthweight < tenthth percentile	2153 (12.6%)	63,826 (9.3%)	< 0.001	1.41 (1.35–1.48)	1.18 (1.12–1.24)
Birthweight > 97th percentile	237 (1.4%)	15,679 (2.3%)	< 0.001	0.60 (0.53–0.69)	0.75 (0.65–0.85)

*BMI* body mass index, *IVF* in vitro fertilization, *PROM* premature rupture of membranes

## Discussion

The main novel finding of our study was that the risk associations increase with each gestational age group after 28 weeks of gestation. With the exception of PPROM, the extremely preterm breech deliveries have similar clinical risk profiles as in cephalic presentation when matched for gestational age. However, as gestation proceeds, the risks start to cluster. In moderate to late preterm pregnancies as in term pregnancies, the breech presentation is a high-risk state being associated with several risk factors: PPROM, oligohydramnios, advanced maternal age, nulliparity, previous cesarean section, fetal birth weight below the tenth percentile, female sex, and fetal congenital anomalies. These are in line with the findings of previous studies [3, 5, 7, 8], that associated breech presentation at term with obstetric risk factors. The prevalence of breech presentation was negatively correlated with the gestational age with a decline from 23.5% in extremely preterm pregnancies to 2.5% at term. The prevalence of breech presentation in preterm pregnancies observed in our trial is similar to that of comparable studies [1, 2].

In extremely preterm deliveries, PPROM was the only risk factor for breech presentation and it stayed as a risk for breech presentation through the gestational weeks. This finding is comparable to the previous literature suggesting that PPROM occurs more often at earlier gestational age in pregnancies with the fetus in breech presentation compared with cephalic [21, 22]. PPROM might prevent the fetus to change into cephalic presentation. Furthermore, Goodman and colleagues (2013) reported that in pregnancies with a fetus in a presentation other than cephalic had more complications such as oligohydramnios, infections, placental abruption, and even stillbirths. In our study, surprisingly, placental abruption seemed to have a negative correlation with breech presentation among extremely preterm deliveries. This inconsistency between our results and the literature might be due to the small number of cases. Many of the obstetric complications, for example gestational diabetes, late pre-eclampsia, and late intrauterine growth restriction develop during the second or the third trimester of the pregnancy which explains partially why the risk factors for breech presentation are rarer in extremely preterm deliveries.

In very preterm delivery, breech presentation was associated with PPROM, pre-eclampsia, and fetal birth weight below the tenth percentile. Fetal growth restriction is a known risk factor for breech presentation at term, since it is associated with reduced fetal movements due to diminished resources [23–25]. Furthermore, fetal growth restriction is known to be the single largest factor for stillbirth and neonatal mortality [26–30]. Maternal arterial hypertension disturbs placental function which might cause low birth weight [31, 32]. Arterial hypertension and pre-eclampsia increased the risk for breech presentation in very preterm births, but not in earlier or later preterm pregnancies. This finding may be due to the bias that pre-eclampsia is a well-described risk factor for PPROM, fetal growth restriction, and preterm deliveries which are also independent markers for breech presentation itself [4, 5, 31, 33, 34]. The severity of early pre-eclampsia might affect the fetal wellbeing, reduce fetal movements and growth, which might reduce the spontaneous fetal rotation to the cephalic position [35]. In addition, the most severe cases might not reach older gestational age before the delivery.

The risk factor for breech presentation in moderate to late preterm breech delivery was PPROM, oligohydramnios, advanced maternal age, nulliparity, previous cesarean section, fetal birth weight below the tenth percentile, female sex, and fetal congenital anomalies. Oligohydramnios is a known significant risk factor for term breech pregnancies [25] and it is linked to the reduced fetal movements partly due to a restricted intrauterine space [24, 35] and nuchal cords [35]. Additionally, oligohydramnios is associated with placental dysfunction, which might reduce fetal resources and thus has a progressive effect on the fetal movements and prevent the fetus from turning into cephalic presentation [3, 4, 18]. Fetal female sex in moderate to late preterm breech pregnancies remained as a risk factor, as identified previously for term pregnancies [3–5]. It has been debated whether this risk is due to a smaller fetal size or that female fetuses tend to move less [9, 20]. The mothers of infants born in breech presentation in moderate to late preterm and term and post-term pregnancies seemed to be older and had an increased risk of having a fetus with a congenital anomaly. The advanced maternal age is associated with negative effects on vascular health, which may have an influence on the developing fetus and increase the incidence of congenital anomalies [19, 34, 36]. Furthermore, congenital anomalies may have a negative influence on fetal movements [19, 35]. Whereas, the low birth weight was found as a risk for breech presentation, a birth weight above the 97th percentile was, coherently a protective factor for breech presentation in very to term and post-term pregnancies.

We found that in term pregnancies, breech presentation was associated with advanced maternal age, nulliparity, maternal hypothyroidism, pre-gestational diabetes, placenta praevia, PROM, oligohydramnios, fetal congenital anomaly, female sex of the fetus, and birth weight below the tenth percentile. A previous cesarean section is known to be positively related to the odds of having a fetus in breech presentation at term [5, 14], and in our study, this risk factor started to show already in moderate to late preterm pregnancies. Instead of the scar being the cause of breech presentation, it is more likely that the women with a history of breech cesarean section have, during subsequent pregnancies, a fetus in breech presentation again or have a cesarean section for another reason [3, 5, 37]. Our data suggest that the advanced maternal age and nulliparity are the risks for breech presentation at term, but as well as in moderate to late preterm pregnancies. The tight wall of the abdomen and the uterus of nulliparous women might inhibit the fetus from rotating to cephalic presentation [9]. In a meta-analysis from 2017, older maternal age has been considered to increase the risk of placental dysfunction such as pre-eclampsia and preterm birth [36] that are also common risk factors for breech presentation [4, 5]. Bearing the first child in older maternal age and giving birth by cesarean section may affect the decision not to have another child and might explain the higher rate of nulliparity among moderate to late preterm and term deliveries [1]. Our study found correlation between maternal hypothyroidism and breech presentation at term. Some studies have demonstrated an association between maternal thyroid hypofunction and adverse pregnancy outcomes such as pre-eclampsia and low birth weight which are, furthermore, risks for breech presentation and may explain partly the higher prevalence of maternal hypothyroidism in term breech deliveries [38–40]. However, the absence of screening of, for example, thyroid diseases may cause bias in the diagnoses.

Our study demonstrated that as gestation proceeds, more obstetric risk factors can be found associating with breech presentation. In the earlier gestation and excluding PPROM, breech deliveries did not differ in obstetric risk factors compared to cephalic. The risk factors in 32 weeks of gestational age are comparable to those in term pregnancy, and several of these factors, such as low birth weight, congenital anomalies and history of cesarean section, are associated with adverse fetal outcomes [1, 4, 5, 8, 14, 17] and must be taken into account when treating breech pregnancies. Risk factors should be evaluated prior to offering a patient an external cephalic version, as the presence of some of these risks may increase the change of failed version or fetal intolerance of the procedure. This study had adequate power to show differences between the risk profiles of breech and cephalic presentations in different gestational phase. Further research, however, is needed for improving the identification of patients at risk for preterm breech labor and elucidating the optimal route for delivery in preterm breech pregnancies.

Our study is unique since it is the first study, to our knowledge, that compares the risks for breech presentation in preterm and term deliveries. The analysis is based on a large nationwide population, which is the major strength of our study. The study population included nearly 34,000 preterm births over 11 years in Finland and 737,788 deliveries overall. The medical treatment of pregnancies is homogenous, since there are no private hospitals treating deliveries. A further strength relates to the important information on the characteristics of the mother, for example smoking during pregnancy and pre-pregnancy body mass index. The retrospective approach is a limitation of the study, another one is the design as a record linkage study, due to which the variables were restricted to the data availability. Therefore, we were not able to assess, for example uterine anomalies or previous breech deliveries to the analysis.

## Conclusion

Our results show that the factors associated with breech presentation in very late preterm breech deliveries resemble those in term pregnancies. However, breech presentation in extremely preterm breech birth has similar clinical risk profiles as in cephalic presentation.

## Abbreviations

ICD-10: International Statistical Classification of Diseases and Related Health Problems 10th Revision
WHO: World Health Organization; BMI, body mass index
PPROM: Preterm premature rupture of membranes
OR: Crude odds ratio
Cl: Confidence interval
aOR: Adjusted odds ratio
PROM: Premature rupture of membranes
